# *N*,*N*′-Bis(2-iodo­benzyl­idene)ethane-1,2-diamine

**DOI:** 10.1107/S1600536808027608

**Published:** 2008-09-06

**Authors:** Hoong-Kun Fun, Reza Kia

**Affiliations:** a X-ray Crystallography Unit, School of Physics, Universiti Sains Malaysia, 11800 USM, Penang, Malaysia

## Abstract

The mol­ecule of the title Schiff base compound, C_16_H_14_I_2_N_2_, lies across a crystallographic inversion centre. An intra­molecular C—H⋯I hydrogen bond forms a five-membered ring, producing an *S*(5) ring motif. The C=N bond is coplanar with the benzene ring and adopts a *trans* configuration. Within the mol­ecule, the planar units are parallel, but extend in opposite directions from the dimethyl­ene bridge. An inter­esting feature of the crystal structure is the short I⋯N [3.2096 (15) Å] inter­action, which is significantly shorter than the sum of the van der Waals radii of these atoms. In the crystal structure, mol­ecules are linked into one-dimensional extended chains along the *c* axis and also into one-dimensional extended chains along the *b* axis through short inter­molecular I⋯N inter­actions, forming two-dimensional networks parallel to the *bc* plane.

## Related literature

For bond-length data, see: Allen *et al.* (1987 ▶). For hydrogen-bond motifs, see: Bernstein *et al.* (1995 ▶). For the hydrogen bond capability of halogens, see: Brammer *et al.* (2001 ▶). For halogen–electronegative atom inter­actions, see: Lommerse *et al.* (1996 ▶). For related structures, see, for example: Fun, Kia & Kargar (2008 ▶); Fun, Kargar & Kia (2008 ▶); Fun, Mirkhani *et al.* (2008 ▶); Calligaris & Randaccio, (1987 ▶). For information on Schiff base ligands, their complexes and their applications, see, for example: Pal *et al.* (2005 ▶); Hou *et al.* (2001 ▶); Ren *et al.* (2002 ▶).
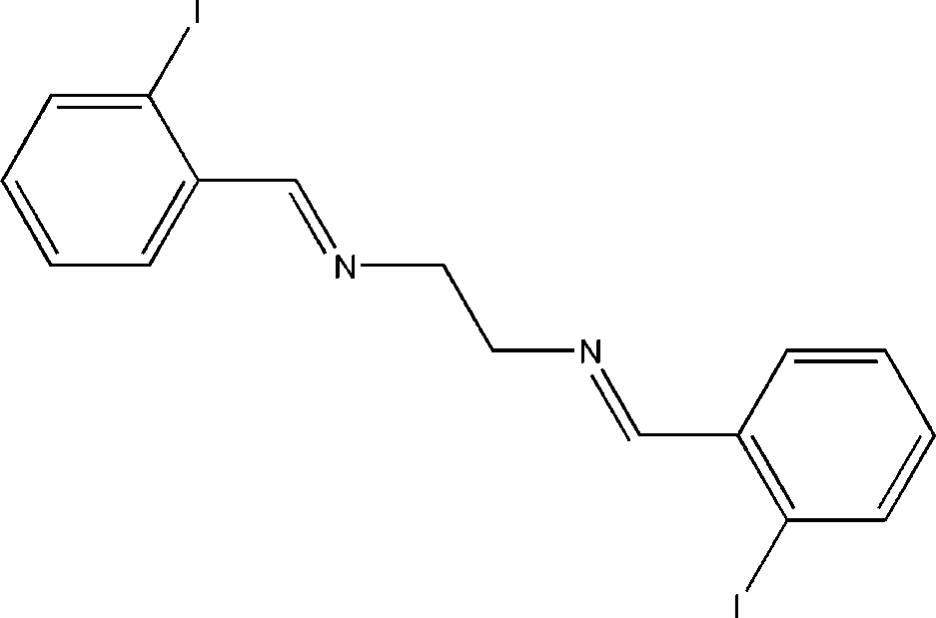


## Experimental

### 

#### Crystal data

C_16_H_14_I_2_N_2_
*M* *_r_* = 488.09
Monoclinic, 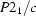

*a* = 12.1820 (4) Å
*b* = 4.5978 (1) Å
*c* = 14.5664 (4) Å
β = 94.424 (2)°
*V* = 813.44 (4) Å^3^
*Z* = 2
Mo *K*α radiation
μ = 3.86 mm^−1^
*T* = 100.0 (1) K
0.51 × 0.14 × 0.02 mm

#### Data collection

Bruker SMART APEXII CCD area-detector diffractometer
Absorption correction: multi-scan (*SADABS*; Bruker, 2005 ▶) *T*_min_ = 0.244, *T*_max_ = 0.916
24819 measured reflections
4235 independent reflections
3466 reflections with *I* > 2σ(*I*)
*R*_int_ = 0.044

#### Refinement

*R*[*F* ^2^ > 2σ(*F* ^2^)] = 0.030
*wR*(*F* ^2^) = 0.074
*S* = 1.16
4235 reflections
115 parameters
All H-atom parameters refined
Δρ_max_ = 1.89 e Å^−3^
Δρ_min_ = −1.74 e Å^−3^

### 

Data collection: *APEX2* (Bruker, 2005 ▶); cell refinement: *APEX2*; data reduction: *SAINT* (Bruker, 2005 ▶); program(s) used to solve structure: *SHELXTL* (Sheldrick, 2008 ▶); program(s) used to refine structure: *SHELXTL*; molecular graphics: *SHELXTL*; software used to prepare material for publication: *SHELXTL* and *PLATON* (Spek, 2003 ▶).

## Supplementary Material

Crystal structure: contains datablocks global, I. DOI: 10.1107/S1600536808027608/at2623sup1.cif

Structure factors: contains datablocks I. DOI: 10.1107/S1600536808027608/at2623Isup2.hkl

Additional supplementary materials:  crystallographic information; 3D view; checkCIF report

## Figures and Tables

**Table 1:** Hydrogen-bond geometry (Å, °)

*D*—H⋯*A*	*D*—H	H⋯*A*	*D*⋯*A*	*D*—H⋯*A*
C7—H7⋯I1	0.93 (3)	2.87 (3)	3.3880 (18)	116 (2)

**Fig. 1.**
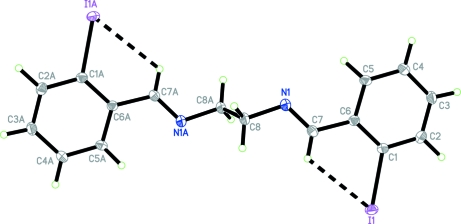
The molecular structure of (I) with atom labels and 50% probability ellipsoids for non-H atoms [symmetry code for A: -x, 1 - y, -z].

**Fig. 2.**
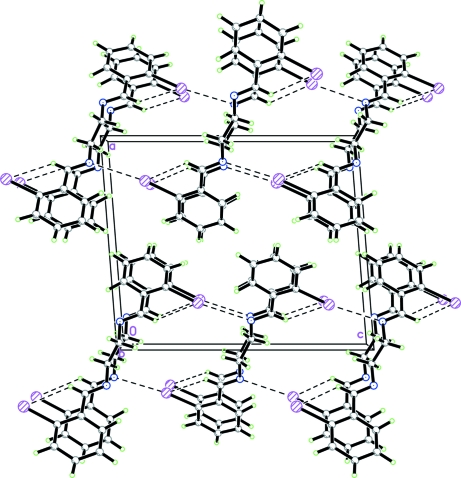
The crystal packing of (I), viewed down the b axis, showing 1-D extended chains along the c axis. Intra- and intermolecular interactions are shown as dashed lines.

**Fig. 3.**
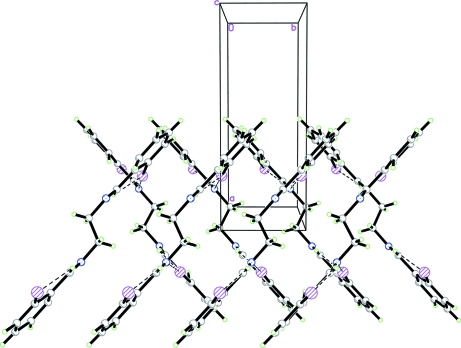
The crystal packing of (I), viewed down the c-axis showing 1-D extended chains along the b-axis. Intra and intermolecular interactions are shown as dashed lines.

## supplementary crystallographic information

### Comment

Schiff bases are one of most prevalent mixed-donor ligands in the field of coordination chemistry. Schiff bases have been used widely as ligands in the formation of transition metal complexes. Many such complexes have been structurally characterized, but only a relatively small number of free Schiff base ligands have been characterized (Calligaris & Randaccio, 1987). There has been growing interest in Schiff base ligands, mainly because of their wide application in the field of biochemistry, synthesis, and catalysis (Pal *et al.*, 2005; Hou *et al.*, 2001; Ren *et al.*, 2002). As an extension of our work (Fun, Kia & Kargar 2008; Fun, Kargar & Kia 2008; Fun, Mirkhani *et al.* 2008) on the structural characterization of Schiff base compounds, the title compound (I), is reported here.

The molecule of the title compound, (I), (Fig. 1), lies across a crystallographic inversion centre. The bond lengths and angles are within normal ranges (Allen *et al.*,1987). An intramolecular C—H···I hydrogen bond (Brammer *et al.* 2001) forms a five-membered ring, producing an *S*(5) ring motif (Bernstein *et al.*, 1995) (Table 1). The asymmetric unit of the compound is composed of one-half of the molecule. The C═N bond is coplanar with the benzene ring and adopts a *trans* configuration. Within the molecule, the planar units are parallel, but extend in opposite directions from the methylene bridge. The interesting feature of the crystal structure is the short I···N [3.2096 (15) Å] interactions (Lommerse *et al.* 1996), which is significantly shorter than the sum of the van der Waals radii of the relevant atoms. In the crystal structure, molecules are linked into 1-D extended chains along the *c* axis and are also into 1-D extended chains along the *b* axis through short intermolecular I···N interactions forming 2-D networks (Fig. 2 & 3) which are parallel to the *bc* plane.

### Experimental

The synthetic method has been described earlier (Fun, Kia & Kargar *et al.*, 2008). Single crystals suitable for *X*-ray diffraction were obtained by evaporation of an ethanol solution at room temperature.

### Refinement

All of the H atoms were located from the difference Fourier map and freely refined. The highest peak is located 0.61 Å from C5 and the deepest hole is located 0.63 Å from I1.

### Crystal data

**Table:**

C_16_H_14_I_2_N_2_	*F*(000) = 460
*M**_r_* = 488.09	*D*_x_ = 1.993 Mg m^−^^3^
Monoclinic, *P*2_1_/*c*	Mo *K*α radiation, λ = 0.71073 Å
Hall symbol: -P 2ybc	Cell parameters from 7125 reflections
*a* = 12.1820 (4) Å	θ = 2.8–38.9°
*b* = 4.5978 (1) Å	µ = 3.86 mm^−^^1^
*c* = 14.5664 (4) Å	*T* = 100 K
β = 94.424 (2)°	Plate, colourless
*V* = 813.44 (4) Å^3^	0.51 × 0.14 × 0.02 mm
*Z* = 2	

### Data collection

**Table:**

Bruker SMART APEXII CCD area-detector diffractometer	4235 independent reflections
Radiation source: fine-focus sealed tube	3466 reflections with *I* > 2σ(*I*)
graphite	*R*_int_ = 0.044
φ and ω scans	θ_max_ = 37.5°, θ_min_ = 1.7°
Absorption correction: multi-scan (SADABS; Bruker, 2005)	*h* = −19→20
*T*_min_ = 0.244, *T*_max_ = 0.917	*k* = −7→7
24819 measured reflections	*l* = −24→24

### Refinement

**Table:**

Refinement on *F*^2^	Primary atom site location: structure-invariant direct methods
Least-squares matrix: full	Secondary atom site location: difference Fourier map
*R*[*F*^2^ > 2σ(*F*^2^)] = 0.031	Hydrogen site location: inferred from neighbouring sites
*wR*(*F*^2^) = 0.074	All H-atom parameters refined
*S* = 1.16	*w* = 1/[σ^2^(*F*_o_^2^) + (0.0295*P*)^2^ + 0.1458*P*] where *P* = (*F*_o_^2^ + 2*F*_c_^2^)/3
4235 reflections	(Δ/σ)_max_ = 0.001
115 parameters	Δρ_max_ = 1.89 e Å^−^^3^
0 restraints	Δρ_min_ = −1.74 e Å^−^^3^

### Special details

**Table:**

Experimental. The low-temperature data was collected with the Oxford Cyrosystem Cobra low-temperature attachment.
Geometry. All e.s.d.'s (except the e.s.d. in the dihedral angle between two l.s. planes) are estimated using the full covariance matrix. The cell e.s.d.'s are taken into account individually in the estimation of e.s.d.'s in distances, angles and torsion angles; correlations between e.s.d.'s in cell parameters are only used when they are defined by crystal symmetry. An approximate (isotropic) treatment of cell e.s.d.'s is used for estimating e.s.d.'s involving l.s. planes.
Refinement. Refinement of *F*^2^ against ALL reflections. The weighted *R*-factor *wR* and goodness of fit *S* are based on *F*^2^, conventional *R*-factors *R* are based on *F*, with *F* set to zero for negative *F*^2^. The threshold expression of *F*^2^ > σ(*F*^2^) is used only for calculating *R*-factors(gt) *etc*. and is not relevant to the choice of reflections for refinement. *R*-factors based on *F*^2^ are statistically about twice as large as those based on *F*, and *R*- factors based on ALL data will be even larger.

### Fractional atomic coordinates and isotropic or equivalent isotropic displacement parameters (Å^2^)

**Table:**

	*x*	*y*	*z*	*U*_iso_*/*U*_eq_	
I1	0.219874 (11)	0.02241 (3)	0.328844 (8)	0.01748 (5)	
N1	0.14038 (12)	0.3597 (4)	0.03053 (10)	0.0158 (3)	
C1	0.29294 (15)	−0.0875 (4)	0.20654 (12)	0.0146 (3)	
C2	0.37697 (15)	−0.2923 (4)	0.21484 (12)	0.0167 (3)	
C3	0.43327 (16)	−0.3648 (5)	0.13879 (12)	0.0178 (3)	
C4	0.40419 (16)	−0.2345 (5)	0.05442 (13)	0.0185 (4)	
C5	0.32010 (17)	−0.0341 (4)	0.04599 (13)	0.0160 (3)	
H5	0.2813	−0.0060	−0.0162	0.019*	
C6	0.26222 (16)	0.0456 (4)	0.12178 (13)	0.0135 (3)	
C7	0.17398 (15)	0.2630 (4)	0.10940 (12)	0.0147 (3)	
C8	0.05021 (16)	0.5681 (4)	0.02623 (13)	0.0160 (3)	
H8B	0.0723 (18)	0.725 (5)	−0.0044 (15)	0.016 (6)*	
H4	0.4465 (18)	−0.278 (5)	0.0004 (15)	0.017 (6)*	
H8A	0.0293 (19)	0.629 (6)	0.0866 (16)	0.021 (6)*	
H2	0.3998 (18)	−0.387 (6)	0.2766 (16)	0.015 (6)*	
H7	0.144 (2)	0.323 (7)	0.1634 (18)	0.034 (7)*	
H3	0.488 (3)	−0.507 (5)	0.148 (2)	0.031 (9)*	

### Atomic displacement parameters (Å^2^)

**Table:**

	*U*^11^	*U*^22^	*U*^33^	*U*^12^	*U*^13^	*U*^23^
I1	0.02070 (7)	0.01995 (7)	0.01214 (6)	0.00044 (4)	0.00353 (4)	0.00024 (4)
N1	0.0157 (7)	0.0154 (8)	0.0163 (6)	0.0034 (6)	0.0008 (5)	0.0003 (5)
C1	0.0162 (8)	0.0149 (8)	0.0127 (7)	−0.0002 (6)	0.0015 (6)	−0.0012 (6)
C2	0.0184 (8)	0.0151 (8)	0.0160 (7)	0.0003 (7)	−0.0026 (6)	0.0001 (6)
C3	0.0155 (8)	0.0175 (9)	0.0201 (8)	0.0046 (7)	−0.0003 (6)	−0.0004 (7)
C4	0.0192 (8)	0.0187 (9)	0.0178 (8)	0.0049 (7)	0.0033 (6)	−0.0019 (6)
C5	0.0188 (8)	0.0161 (8)	0.0132 (7)	0.0018 (6)	0.0028 (6)	−0.0030 (6)
C6	0.0151 (8)	0.0132 (8)	0.0123 (7)	0.0001 (6)	0.0011 (6)	−0.0011 (6)
C7	0.0147 (7)	0.0129 (8)	0.0168 (7)	0.0008 (6)	0.0021 (6)	−0.0011 (6)
C8	0.0151 (8)	0.0149 (8)	0.0178 (8)	0.0038 (6)	0.0009 (6)	0.0002 (6)

### Geometric parameters (Å, °)

**Table:**

I1—C1	2.1133 (17)	C4—C5	1.376 (3)
N1—C7	1.270 (2)	C4—H4	0.99 (2)
N1—C8	1.455 (2)	C5—C6	1.404 (3)
C1—C2	1.389 (3)	C5—H5	0.9975
C1—C6	1.403 (3)	C6—C7	1.469 (3)
C2—C3	1.388 (3)	C7—H7	0.93 (3)
C2—H2	1.02 (2)	C8—C8^i^	1.526 (4)
C3—C4	1.388 (3)	C8—H8B	0.90 (2)
C3—H3	0.94 (3)	C8—H8A	0.98 (2)
			
C7—N1—C8	117.33 (16)	C4—C5—H5	117.7
C2—C1—C6	121.13 (17)	C6—C5—H5	116.7
C2—C1—I1	116.36 (13)	C1—C6—C5	117.54 (18)
C6—C1—I1	122.46 (14)	C1—C6—C7	123.23 (17)
C3—C2—C1	119.97 (17)	C5—C6—C7	119.23 (17)
C3—C2—H2	118.9 (13)	N1—C7—C6	122.09 (17)
C1—C2—H2	121.1 (13)	N1—C7—H7	122.8 (17)
C2—C3—C4	119.67 (18)	C6—C7—H7	115.1 (17)
C2—C3—H3	116 (2)	N1—C8—C8^i^	108.9 (2)
C4—C3—H3	124 (2)	N1—C8—H8B	107.1 (14)
C5—C4—C3	120.34 (18)	C8^i^—C8—H8B	109.9 (14)
C5—C4—H4	119.5 (13)	N1—C8—H8A	113.6 (15)
C3—C4—H4	120.1 (13)	C8^i^—C8—H8A	108.3 (14)
C4—C5—C6	121.35 (18)	H8B—C8—H8A	109 (2)
			
C6—C1—C2—C3	1.1 (3)	I1—C1—C6—C7	−2.6 (3)
I1—C1—C2—C3	−176.50 (15)	C4—C5—C6—C1	−0.2 (3)
C1—C2—C3—C4	−0.9 (3)	C4—C5—C6—C7	179.31 (18)
C2—C3—C4—C5	0.1 (3)	C8—N1—C7—C6	178.18 (17)
C3—C4—C5—C6	0.5 (3)	C1—C6—C7—N1	−173.04 (19)
C2—C1—C6—C5	−0.5 (3)	C5—C6—C7—N1	7.4 (3)
I1—C1—C6—C5	176.90 (13)	C7—N1—C8—C8^i^	−114.6 (2)
C2—C1—C6—C7	179.94 (17)		

Symmetry codes: (i) −*x*, −*y*+1, −*z*.

### Hydrogen-bond geometry (Å, °)

**Table:**

*D*—H···*A*	*D*—H	H···*A*	*D*···*A*	*D*—H···*A*
C7—H7···I1	0.93 (3)	2.87 (3)	3.3880 (18)	116 (2)
